# Comparative Analysis of Microbiota in Jiani Yaks with Different Rib Structures

**DOI:** 10.3390/life14111458

**Published:** 2024-11-10

**Authors:** Yangji Cidan, Sijia Lu, Hongzhuang Wang, Jia Wang, Munwar Ali, Dalia Fouad, Farid S. Ataya, Yanbin Zhu, Wangdui Basang, Kun Li

**Affiliations:** 1 State Key Laboratory of Hulless Barley and Yak Germplasm Resources and Genetic Improvement, Lhasa 850002, China; 13889092363@163.com (Y.C.); wanghongzhuang66@163.com (H.W.); bw0891@163.com (W.B.); 2 Institute of Animal Husbandry and Veterinary Medicine, Tibet Academy of Agriculture and Animal Husbandry Science, Lhasa 850009, China; 3 College of Veterinary Medicine, Nanjing Agricultural University, Nanjing 210095, China; 2024107091@stu.njau.edu.cn (S.L.); 2024207034@stu.njau.edu.cn (J.W.); drmunwarali06@gmail.com (M.A.); 4 Department of Zoology, College of Science, King Saud University, P.O. Box 22452, Riyadh 11495, Saudi Arabia; fouaddalia385@gmail.com; 5 Department of Biochemistry, College of Science, King Saud University, P.O. Box 2455, Riyadh 11451, Saudi Arabia; faridataya385@gmail.com

**Keywords:** yak, Jiani, rib structure, rumen, microbiota, sequencing

## Abstract

The Jiani yak is a nationally renowned species that is known for its meat which is rich in various minerals, amino acids, and proteins. The rumen microbiota plays a critical role in gastrointestinal health and feed degradation, contributing proteins, lipids, and volatile fatty acids (VFAs) essential for milk and meat production. However, there is limited knowledge about the microbiota of free-ranging Jiani yaks, especially those with 15 ribs. Rumen fluid samples were collected from yaks with 14 (PL) ribs and 15 (DL) ribs from a slaughterhouse in Jiani County, China. The total DNA of rumen fluid microorganisms was extracted for microbiota sequencing. Our results revealed 643,713 and 656,346 raw sequences in DL and PL animals, respectively, with 611,934 and 622,814 filtered sequences in these two yak groups. We identified 13,498 Amplicon Sequence Variants (ASVs), with 2623 shared between DL and PL animals. The ratio of Bacteroidota to Firmicutes differed between PL (3.04) and DL (2.35) animals. Additionally, 6 phyla and 21 genera showed significant differences between yaks with 14 and 15 ribs, leading to altered microbiota functions, with 51 and 35 notably different MetaCyc and KEGG pathways, respectively. Hence, the microbiota of yaks with 15 ribs differs from those with 14 ribs. Therefore, these microbiota-related comparative investigations will provide insights into yak husbandry practices and genetic selection strategies for their improved productivity in harsh environments.

## 1. Introduction

The yak is a distinctive bovine species native to the plateau, renowned for its long and dense hair [1,2]. This animal is of significant economic importance in the plateau region, providing meat, milk, horn, and fur for the native Tibetan population [3]. In addition to these resources, yaks are commonly used for transportation, and their excrement serves as fuel in cold plateau environments [4]. Jiani County, located in the southeast of Naqu, Xizang, China, spans from 91°09′ to 94°01′ E longitude and from 31°07′ to 32°00′ N latitude. The average altitude of Jiani County exceeds 4500 m, with an annual temperature averaging −0.21 °C. The Yak industry is a central and vital economic industry in Jiani Country, with over 120,000 yaks reported in this region as of 2020. Jiani yaks are notable for their nutritionally rich meat and milk [5]. Therefore, exploring the gut microbiota of Jiani yaks in a comparative way will lead to the development of strategies for their better production and reproduction, and ultimately lead to benefits to the public health sector in the form of better production and returns.

Among Jiani yaks, most of the yaks have 14 rib structures and only a small proportion possess 15 ribs [6]. This difference in rib structure is attributed to their genetics, which is not only responsible for their skeletal difference but also affects their physiological growth and development [7]. Yaks with 14-rib structures have noticeable humps over their shoulders, their limbs are comparatively shorter, providing stability in rugged terrains, and their thick, long hairs are typically black or brown. The chest of 15-rib structure yaks is expansive, their hump is more prominent, and their longer limbs provide better suitability. Although Jiani yaks with 15-rib structures are fewer, 52% of Jinchuan yaks have a 15-rib structure [8,9]. Yaks in Jiani Country that have a 15-rib structure produce milk with comparatively higher content of essential amino acids and minerals, e.g., calcium and selenium [7]; also, the meat of 15 rib yaks is better in quality and quantity due to their faster growth rate [7,10], which can be correlated with their increased metabolic demands and better feed utilization efficiency. Similarly, higher reproductive efficiency can also be correlated to body size with better outcomes in the 15-rib yaks [10], but it is influenced by other factors like nutritional composition and environmental conditions, which ultimately shape gut microbiota.

The mammalian digestive system is recognized as an important microbial ecological system, serving various functions such as converting ingested feed into nutrition for the host through interaction with its colonized microorganisms [11,12]. Gut microbiota comprises numerous microorganisms like bacteria, archaea, fungi, viruses, and protists [13]. These microbial populations can influence the host’s health by affecting digestion, metabolism, neurological functions, and immunity [14,15]. The rumen of ruminants is a typical ecosystem that plays vital roles in plant digestion [16] and maintains host physiology [17]. The rumen, a crucial digestive organ and fermentation chamber, is home to millions of microorganisms, including bacteria, ciliate protozoa, anaerobic fungi, phages, and methanogens [18,19,20]. The rumen microbiota plays a role in providing proteins, lipids, and VFAs necessary for the production of milk and meat [21,22]. However, an imbalance in the rumen microbial flora, coupled with conditions such as sub-acute ruminal acidosis and mastitis, can adversely affect these processes [23,24]. Along with other physiological parameters, the rumen microbiota is influenced by the host’s genetics [25], and it is also affected by environmental factors, forage composition, and age [2,26,27].

Despite the established importance of yaks’ gut microbiota in the host’s adaptation to harsh environments, limited studies have explored how variable rib structures influence microbial composition and functions which are important for the health and, ultimately, the performance of the animals [28,29,30]. Keeping in view the significant physiological differences in yaks with different rib structures, we conducted this research to compare the microbiota characteristics of Jiani yaks with different rib configurations. To the best of our knowledge, very limited information is available regarding comparative variations in microbiota with different rib structures [29,31]. This research will lead to better yak breeding programs to select for superior rib structures and provide insights into the role of microbiota in yak health and ultimately performance and policy development, emphasizing the importance of genetic diversity in livestock management for food security and rural development. Therefore, revealing the microbiota characteristics of yaks is meaningful and helpful for scientific farming.

## 2. Materials and Methods

### 2.1. Experimental Design

**Figure 1:**
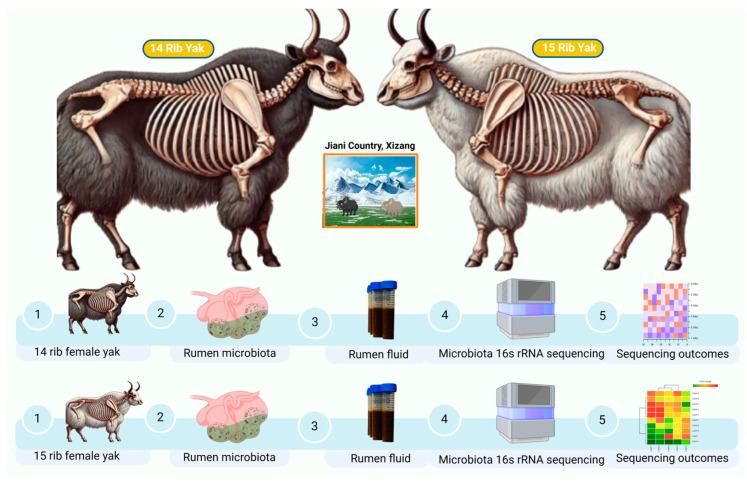
Experimental design: sample collection to compare the rumen microbiota of 14- and 15-rib yaks.

Rumen fluid samples were collected from a total of 12 4-year-old female yaks with 14 ribs (PL, n = 6) or 15 ribs (DL, n = 6) at a slaughterhouse in Jiani County, China, in December 2023. All of the yaks included here were grazing yaks from the same herd. The 15-rib-structure yaks comprised 5% and the 14-rib-structure yaks comprised 95% of the herd. During antemortem and post-mortem inspection, no specific lesions were detected, all of the female yaks included were healthy, and no antibiotic treatment was implied before slaughtering. A total of 50mL of rumen fluid was collected from each yak, ensuring that environmental and cross-contamination were eliminated to a maximum extent. The rumen fluids were then stored at −80 °C for further study (Figure 1).

In Jiani County, grazing yaks mostly consume a variety of forages consisting of legumes, grasses, and different types of herbs, to obtain non-dispensable nutrients for their survival [7]. The predominant grasses include species such as *Poa* and *Stipa*, with fiber content typically ranging from 30 to 50% Neutral Detergent Fiber (NDF). Legumes like clover and alfalfa are also significant, providing a crude protein content of about 12–16% [32]. In addition, yaks may eat shrubs and herbs, including *Artemisia*, as a good source of essential minerals. Broadly, the nutritional makeup of grazing yaks contains an energy content of around 2.0–2.5 Mcal/kg dry matter, and a moderate level of vitamins [33].

### 2.2. Ethical Statement

The primary purpose of these yaks was for slaughter to obtain meat and the resulting viscera were abandoned, so there was no ethical issue in using their rumen fluid for the estimation of microbiota variations in yaks with different rib structures.

### 2.3. DNA Extraction, PCR Amplification, and Sequencing via Illumina Novaseq

The total DNA of microbes from rumen fluid was extracted using a MolPure^®^ stool DNA kit (Yeasen, Shanghai, China) according to the manufacturer’s instructions. After extraction, the DNA solution was stored at −20 °C for further analysis. The selected regions for sequencing (V3–V4) were amplified using PCR using specific primers (338F/806R primer pairs (F: 5′-ACTCCTACGGGAGGCAGCA-3′; R: 5′-GGACTACHVGGGTWTCTAAT-3′) [34] with barcode and high-fidelity DNA polymerase. The PCR reaction mixture for each amplification reaction contained 12.5 μL PCR Mix, 1.0 μL DNA, 1.0 μL primer (of each forward and reverse primer), and 9.5 μL dd.H_2_O, with the resultant reaction mixture’s volume being 25 μL. PCR amplification consisted of a total of 35 PCR cycles, with each cycle having an initial hot starting pre-denaturation temperature of 95 °C (5 m), then 95 °C (15 s); the primer annealing temperature Tm was 50 °C (15 s); the elongation at 72 °C (45 s) was followed by extension at 72 °C (10 m), and, finally, storage at 4 °C.

The resulting products were assessed using a UV-vis spectrophotometer NanoDrop ND-1000 (Thermo Scientific, Waltham, MA, USA) and 1.2% agarose gel electrophoresis. Subsequently, the generated products from yaks were purified and quantified using the commercial kits of VAHTSTM DNA clean beads (Vazyme, Nanjing, China) and Quant-iT PicoGreen dsDNA assay (Invitrogen, Waltham, MA, USA). The amplicon products were then subjected to a pair-end 2 sequencing (250 bp) on the Illumina MiSeq platform at Bioyi Biotechnology, Co., Ltd. (Wuhan, China). And a library was constructed. The resultant library was inspected using an Agilent Bioanalyzer 2100 (Agilent Technologies, Santa Clara, CA, USA) and a Promega QuantiFluor (Promega, Madison, WI, USA). Following the qualification of the library, it was sequenced.

### 2.4. 16S rRNA Gene Sequencing and Bioinformatics Analysis

Following sequencing, the raw sequences obtained were filtered and merged for quality control using DADA2 (version: 1.31.0) [35]. The amplicon sequence variants were aligned with MAFFT (version: 7.526), and a Venn graph was created to visualize the shared and unique ASV among different Jiani yak groups by employing the R package (version: 4.3.0) [36]. A taxonomic composition analysis of yaks was conducted using QIIME2 (version: 2023.2) [37]. The richness and evenness of yaks were assessed through ASV-level alpha diversity indices calculation using QIIME2 [38]. The structural variation in yak rumen microbiota across PL and DL animals was explored through a beta diversity analysis including the PCA (principal coordinate analysis), NMDS (nonmetric multidimensional scaling), and unweighted pair-group methods [39,40,41]. Distinctions in bacteria between DL and PL yaks were compared using ZicoSeq, Linear discriminant analysis effect size, and a *t*-test [42]. The microbial functions of yaks were predicted using PICRUSt2 (version: 2.5.0) based on the MetaCyc and KEGG databases [43].

### 2.5. Statistical Analysis

All of the data obtained from Jiani yaks were calculated using Student’s *t*-test with IBM SPSS (26.0). In addition to estimating the statistical difference between the medians of the two groups, a non-parametric statistical test known as the Kruskal–Wallis test was employed. Data are presented as means ± SD, and statistical significance is noted when *p* < 0.05.

## 3. Results

### 3.1. Analysis of Sequencing Data

**Figure 2:**
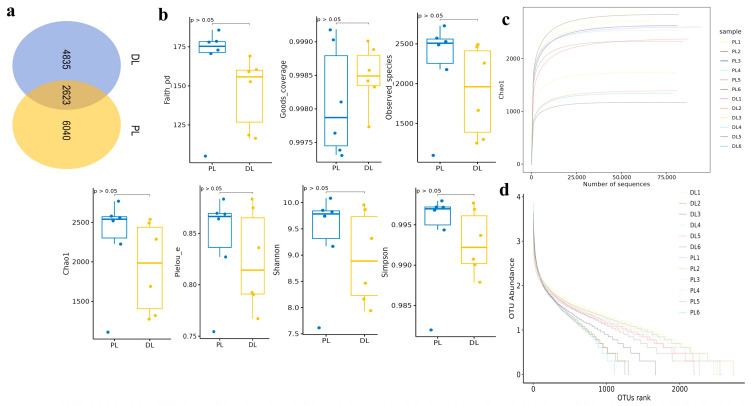
Venn and alpha diversity analysis between 14-rib (PL) and 15-rib (DL) animals. (**a**): Venn map showing ASVs and shared ASV sequences. (**b**): Alpha diversity index. (**c**): Rarefaction curve (flat curves suggesting sufficient sequencing depth). (**d**): Rank abundance curve (broken lines indicating higher microbiota evenness). The different colors represent the different samples.

In yaks with 14-rib structures, over 102,000 raw sequences and 96,900 filtered sequences were generated (Table 1), with the length of the sequenced fragments mostly around 400–430 bp (98.86%). However, in yaks with 15 ribs, there were over 102,000 (DL, PL) raw sequences, while there were 97,000 (DL) and 96,900 (PL) filtered sequences. ASVs were recognized by using DADA2 in QIIME2 for denoising raw reads and conducting de-replication. The sequences were aligned to 13,498 ASVs with 2623 shared ASVs between DL and PL animals, while 4835 and 6040 unique OTUs were found in DL and PL yaks, respectively (Figure 2a). Subsequently, all ASVs were annotated to different taxa (Table 2). The phylum number in both DL and PL yaks ranged from 0 to 2, classes from 35 to 80 in DL yaks and from 31 to 88 in PL yaks, orders from 36 to 53 in DL yaks and from 22 to 69 in PL yaks, families from 227 to 470 in DL yaks and from 201 to 378 in PL yaks, and genus number ranged from 353 to 785 in DL yaks and from 298 to 982 in PL yaks, respectively.

The alpha diversity of the yak gut microbial fraction was calculated to compare the gut microbiota of yaks with 14- and 15-rib structures. The Chao1 estimator was used to describe the richness of a community, while the Shannon index and Simpson index were used to show the diversity of species in the samples. There was no significant difference between the two groups of yaks. The alpha diversity analysis indicated that Faith_pd (PL = 165.11 ± 27.20, DL = 145.64 ± 20.75), Goods_coverage (PL = 0.9981 ± 0.0008, DL = 0.9985 ± 0.0004), Observed_species (PL = 2265.43 ± 546.38, DL = 1906.03 ± 522.26), Chao1 (PL = 2294.53 ± 551.53, DL = 1931.15 ± 531.61), Pielou_e (PL = 0.8445 ± 0.0437, DL = 0.8240 ± 0.0439), Shannon (PL = 9.3822 ± 0.8335, DL = 8.9475 ± 0.8050), and Simpson (PL = 0.9943 ± 0.0056, DL = 0.9928 ± 0.0036) were not significantly different between PL and DL yaks (Table 3), which implies that the microbiota was not changed significantly. There was more diversity in Faith_pd, Observed_species, Chao1, Pielou_e, Shannon, and Simpson in DL yaks compared to PL, but the difference was not significant, whereas diversity in Goods_coverage was less in DL compared to PL yaks; the difference was again non-significant (Figure 2b). The rarefaction curves terminally tending to be horizontal imply that the sequencing depth and scope were adequate for further analysis. Rarefaction curves demonstrated that all yak curves were flat, indicating sufficient sequencing depth in ruminants, and the yak samples were sufficient to reflect the microbiota diversity and were adequate for further analysis (Figure 2c). The rank abundance plot shows the richness and uniformity of each sample. Rank abundance curves were flat, indicating higher microbiota evenness in yaks (Figure 2d).

### 3.2. Ruminal Microbiota Composition of Yaks at Different Taxonomic Levels

**Figure 3:**
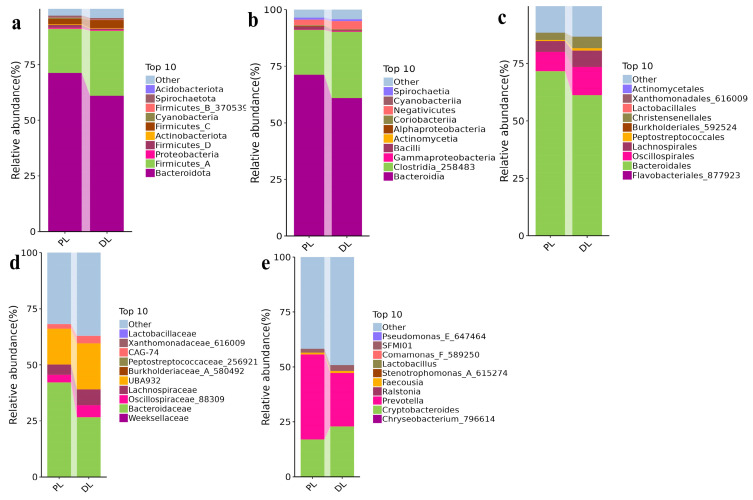
Comparing microbiota structure of 14 rib (PL) and 15 rib (DL) yaks in different taxa. (**a**): Relative abundance of top 10 phyla. (**b**): Relative abundance of top 10 classes. (**c**): Relative abundance of top 10 orders. (**d**): Relative abundance of top 10 families. (**e**): Relative abundance of top 10 genera. The different colors represent the relative abundance of phylae, classes, orders, families, and genera in PL and DL yaks.

At the phylum level, the top 10 phylae were Bacteroidota, Firmicutes_A, Proteobacteria, Firmicutes_D, Actinobacteriota, Firmicutes_C, Cyanobacteria, Firmicutes_B_370539, Spirochaetota, and Acidobacteriota. Among these phyla, Bacteroidota (71.28%), Firmicutes_A (19.76%), Firmicutes_C (2.44%), and Firmicutes_D (1.28%) dominated in PL, whereas Bacteroidota (65.91%), Firmicutes_A (25.30%), Firmicutes_C (2.36%), and Firmicutes_D (0.36%) were predominantly found in DL yaks (Figure 3a). At the class level, the top 10 classes were Bacteroidia, Clostridia_258483, Gammaproteobacteria, Bacilli, Actionomycetia, Alphaproteobacteria, Coriobacteriia, Negativicutes, Cyanobacteria, and Spirochaaetia. Bacteroidia (71.28%), Clostridia_258483 (19.76%), Negativicutes (2.44%), and Bacilli (1.28%) were the predominant classes in PL, while Bacteroidia (60.97%), Clostridia_258483 (29.24%), Negativicutes (3.59%), and Bacilli (0.59%) were the primary classes in DL (Figure 3b). At the order level, the top 10 orders were Flavobacteriales_877923, Bacteroidales, Oscillospirales, Lachnospirales, Peptostreptococcales, Burkholderiales_592524, Christensenellales, Lactobacillales, Xanthomonadales _616009, and Actinomycetales. Bacteroidales (71.64%), Oscillospirales (8.38%), Lachnospirales (4.76%), and Christensenellales (3.09%) were the main orders in PL, while Bacteroidales (61.20%), Oscillospirales (12.21%), Lachnospirales (7.19%), and Christensenellales (4.98%) predominated in DL yaks (Figure 3c). At the family level, the top 10 abundant families were Weeksellaceae, Bacteroidaceae, Oscillospiraceae_88309 Lachnospiraceae, UBA932, Burkholderiaceae_A_580492, Peptostreptococcaceae_256921, CAAG-74, and Xanthomonadaceae_616009. Bacteroidaceae (42.16%), UBA932 (15.91%), Lachnospiraceae (4.63%), and Oscillospiraceae_88309 (3.36%) were primarily detected in PL yaks, while Bacteroidaceae (26.64%), UBA932 (20.55%), Lachnospiraceae (7.09%), and Oscillospiraceae_88309 (5.33%) were the main families in DL yaks (Figure 3d). At the genus level, the most abundant genera were *Chryseobacterium_796614*, *Cryptobacteroides*, *Prevotella*, *Ralstonia*, *Faecousia*, *Stenotrophomonas_A_615274*, *Lactobacillus*, *Comamonas_F_589250*, *SFMI01*, and *Pseudomonas_E_675464*. *Prevotella* (38.69%), *Cryptobacteroides* (16.98%), *SFMI01* (1.73%), and *Faecousia* (0.86%) dominated genera in PL yaks, while *Prevotella* (24.25%), *Cryptobacteroides* (22.97%), *SFMI01* (2.72%), and *Faecousia* (0.87%) were the main genera in DL yaks (Figure 3e).

### 3.3. Comparing Microbiota Structure in Yaks

**Figure 4:**
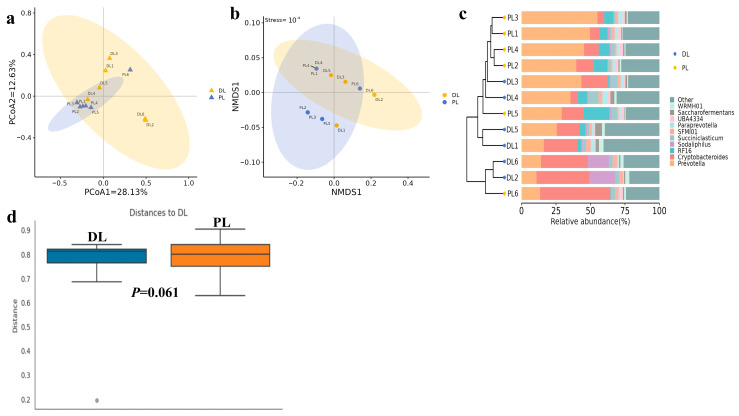
Beta diversity shows the change in microbiota between 14-rib (PL) and 15-rib (DL) yaks. (**a**): Principal coordinate analysis (PCoA). (**b**): Non-metric multidimensional scaling (NMDS) analysis. (**c**): Unweighted pair group method with arithmetic mean (UPGMA) clustering tree. (**d**): Permutational multivariate analysis of variance (PERMANOVA) analysis. The greater distances between samples indicate greater differences in microbial community composition. The different colors represent relative abundance in PL and DL yaks.

Beta diversity analysis was carried out through PCoA, NMDS, UPGMA, and PERMANOVA. Samples with high similarities in community structure tend to cluster together, while samples with significant differences in community structure tend to be far apart. PCoA evaluated the differences and similarities between and within groups, which revealed a distance (PCoA1 = 12.63%; PCoA1 = 28.13%) between PL and DL (Figure 4a). PCoA analysis demonstrated that the distance between sample dots within the group was relatively greater in DL compared to PL yaks, which indicated that DL (15-rib) has a greater effect on ruminal microbiota. The distance between groups (PL and DL) was relatively far, which again indicated the effect of rib structure on microbiota. The NMDS plot (Figure 4b) further supports these findings, which reflected the differences in samples via the distance between dots and stress based on genetic variation, leading to different rib structures. The distance was almost similar among the dots, but it was higher both between the samples within the group and between the DL and PL groups. The NMDS plot also proves that the yaks with different rib structures also have variations in gut microbiota. UPGMA clustering revealed the relationship between the species and abundance of each group at the genus level (Figure 4c). It indicated that the most abundant genera are *Provetella*, *Cryptobacteroides, RF16*, *Sodaliphilus*, *Succiniclasticum*, *SFMI01*, *Paraprevotella*, *UBA4334*, *Saccharofermentans*, and *WRMH01*. The relative abundance of these genera between the samples is shown in Figure 4c. Permanova analysis also supports these findings, which are shown by the distance between the PL and DL groups; however, this is not significant (*p* = 0.061) (Figure 4d).

**Figure 5:**
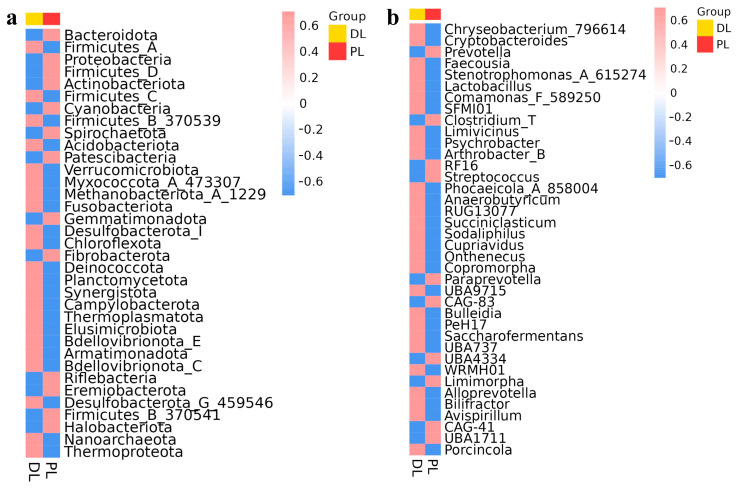
Heatmap analysis of the abundance distribution of the top 50 species between 14-rib (PL) and 15-rib (DL) yak groups. (**a**): Phylum, (**b**): genus.

The heatmap showed that the top phyla were Bacteroidota, Firmicutes_A, Proteobacteria, Firmicutes_D, Actinobacteriota, Firmicutes_C, Cyanobacteria, Firmicutes_B_370539, Spirochaetota, Acidobacteriota, Patescibacteria, Verrucomicrobiota, Myxococcota_A_473307, Methanobacteriota_A _1229, Fusobacteriota, Gemmatimonadota, Desulfobacterota_I, Chloroflexota, Fibrobacterota, Deinococcota, Planctomycetota, Synergistota, Campylobacterota, Thermoplasmatota, Elusimicrobiota, Bdellovibrionota_E, Armatimonadota, Bdellovibrionota_C, Riflebacteria, Eremiobacterota, Desulfobacterota_G_459546, Firmicutes_B_370541, Halobacteriota, Nanoarchaeota, and Thermoproteota. The phyla Bacteroidota and Firmicutes_D were more abundant in PL, whereas Firmicutes_A, Firmicutes_C, and Methanobacteriota_A_1229 were prevalent in DL (Figure 5a). At the genus level, the important genera were *Chryseobacterium_796614*, *Cryptobacteroides*, *Prevotella*, *Faecousia*, *Stenotrophomonas_A_615274*, *Lactobacillus*, *Comamonas_F_589250*, *SFMI01*, *Clostridium_T*, *Limivicinus*, *Psychrobacter*, *Arthrobacter_B*, *RF16*, *Streptococcus*, *Phocaeicola_A_858004*, *Anaerobutyricum*, *RUG13077*, *Succiniclasticum*, *Sodaliphilus*, *Cupriavidus*, *Onthenecuş*, *Copromorpha*, *Paraprevotella*, *UBA9715*, *CAG-83*, *Bulleidia*, *PeH17*, *Saccharofermentans*, *UBA737*, *UBA4334*, *WRMH01*, *Limimorpha*, *Alloprevotella*, *Bilifractor*, *Avispirillum*, *CAG-41*, and *UBA1711*. Genus *Porcincola*, *Prevotella*, *RF16*, *UBA4334*, and *UBA1711* were more abundant in PL, while *Cryptobacteroides*, *Sodaliphilus*, *WRMH01*, and *Saccharofermentans* were found to be abundant in DL group (Figure 5b).

**Figure 6:**
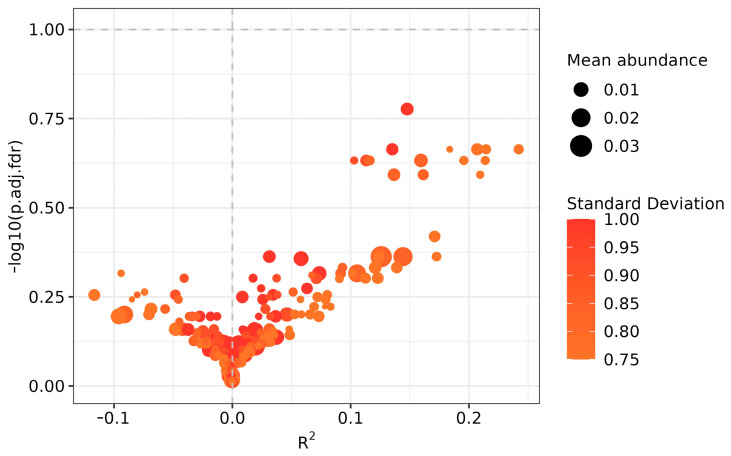
ZicoSeq analysis identified 24 noteworthy ASVs between PL and DL yaks.

ZicoSeq analysis identified 24 noteworthy ASVs between PL and DL yaks, including ASV_1161 (*p* < 0.01), ASV_26571 (*p* < 0.01), ASV_11722 (*p* < 0.01), ASV_11038 (*p* < 0.01), ASV_863 (*p* < 0.01), ASV_17822 (*p* < 0.05), ASV_22832 (*p* < 0.05), ASV_14767 (*p* < 0.05), ASV_33922 (*p* < 0.05), ASV_6943 (*p* < 0.05), ASV_20824 (*p* < 0.05), ASV_30991 (*p* < 0.05), ASV_18946 (*p* < 0.05), ASV_8641 (*p* < 0.05), ASV_14668 (*p* < 0.05), ASV_22063 (*p* < 0.05), ASV_13739 (*p* < 0.05), ASV_9225 (*p* < 0.05), ASV_20458 (*p* < 0.05), ASV_10371 (*p* < 0.05), ASV_35252 (*p* < 0.05), ASV_17378 (*p* < 0.05), and ASV_8426 (*p* < 0.05) (Figure 6).

**Figure 7:**
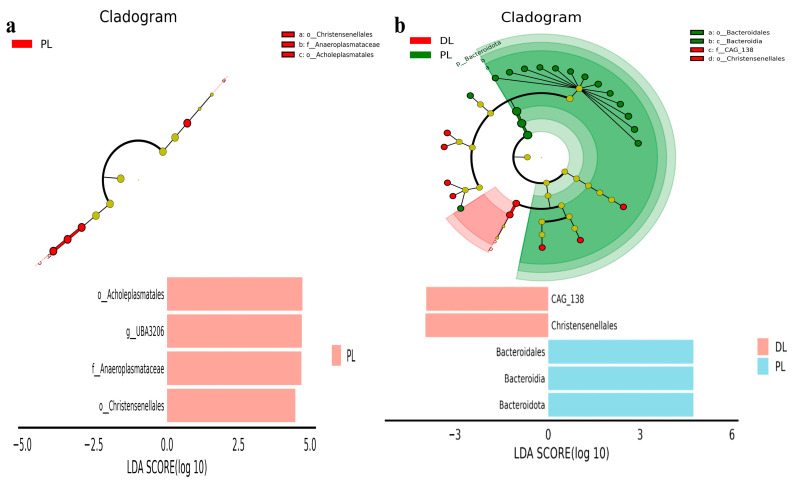
The bacteria distinguished between PL and DL yaks were analyzed using LEfSe. (**a**): Phylum, (**b**): genus. The different colors represent the groups (PL, DL). LDA means linear discriminant analysis. The positive values of the LDA score (log10) indicate the significantly increased relative abundance of taxa, while the negative values of the LDA score (log10) indicate the decreased relative abundance of taxa.

LEfSe analysis was conducted through linear discriminant analysis (LDA) score. The positive values of the LDA score (log10) indicate the significantly increased relative abundance of taxa, while the negative values of the LDA score (log10) indicate the significantly decreased relative abundance of taxa in DL and PL yaks. LEfSe analysis revealed that phyla of Acholeplasmatales, UBA3206, Anaeroplasmataceae, and Christensenellales were more abundant in PL yaks, while there were no prominent phyla in DL yaks (Figure 7a). Genera CAG_138 and Christensenellales were significantly more in DL yaks, while Bacteroidales, Bacteroidia, and Bacteroidota were significantly more abundant in PL yaks (Figure 7b).

**Figure 8:**
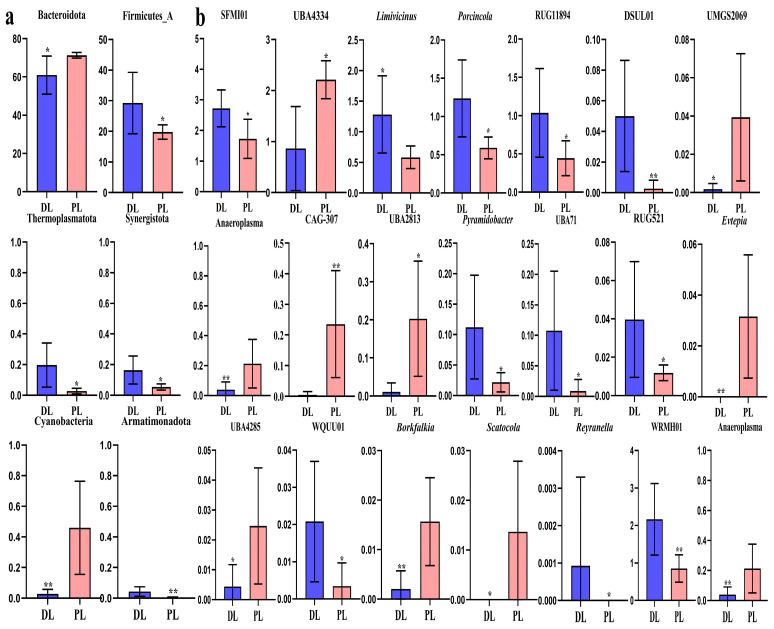
Distinguished bacteria between PL and DL yaks were analyzed using *t*-tests. (**a**): Phylum, (**b**): genus. * *p* < 0.05; ** *p* < 0.01.

The results show that phyla Bacteroidota (*p* < 0.05) and Cyanobacteria (*p* < 0.01) were notably higher in PL (14-rib) yaks, while Firmicutes_A (*p* < 0.05), Thermoplasmatota (*p* < 0.05), Synergistota (*p* < 0.05), and Armatimonadota (*p* < 0.01) were significantly higher in DL (15-rib) yaks (Figure 8a). Genera *SFMI01* (*p* < 0.05), *WRMH01* (*p* < 0.01), *Limivicinus* (*p* < 0.05), *Porcincola* (*p* < 0.05), *RUG11894* (*p* < 0.05), *Pyramidobacter* (*p* < 0.05), *UBA71* (*p* < 0.05), *DSUL01* (*p* < 0.01), *RUG521* (*p* < 0.05), *WQUU01* (*p* < 0.05), and *Reyranella* (*p* < 0.05) were notably higher in DL yaks, while *UBA4334* (*p* < 0.05), *UBA1394* (*p* < 0.05), *Anaeroplasma* (*p* < 0.01), *CAG-307* (*p* < 0.01), *UBA2813* (*p* < 0.05), *UMGS2069* (*p* < 0.05), *Evtepia* (*p* < 0.01), *UBA4285* (*p* < 0.05), *Borkfalkia* (*p* < 0.01), and *Scatocola* (*p* < 0.05) were significantly higher in PL yaks (Figure 8b).

### 3.4. Functional Prediction of Yaks’ Rumen Microbiota

**Figure 9:**
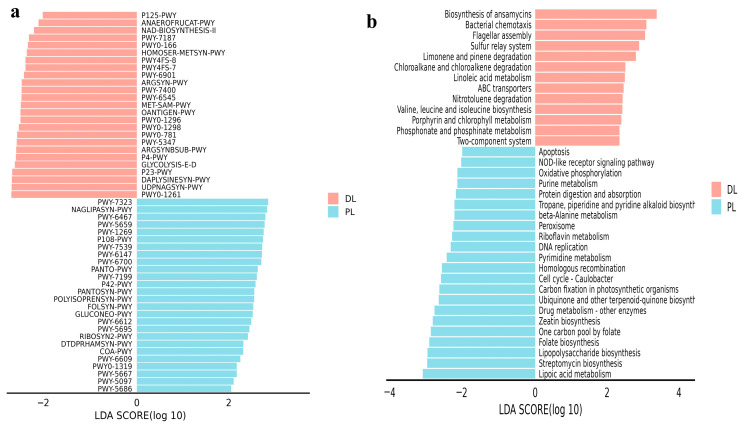
Distinguished pathways between PL and DL yaks. (**a**): MetaCyc pathways, (**b**): KEGG pathways.

The MetaCyc pathways analysis highlighted distinct patterns between PL and DL yaks. Functional prediction via PICRUSt2 revealed 51 significantly different MetaCyc pathways including P125-PWY, ANAEROFRUCAT-PWY, NAD-BIOSYNTHESIS-II, PWY-7187, PWY0-166, HOMOSER-METSYN-PWY, PWY4FS-8, PWY4FS-7, PWY-6901, ARGSYN-PWY, PWY-7400, PWY-6545, MET-SAM-PWY, OANTIGEN-PWY, PWYO-1296, PWYO-1298, PWY0-781, PWY-5347, ARGSYNBSUB-PWY, P4-PWY, GLYCOLYSIS-E-D, P23-PWY, DAPLYSINESYN-PWY, UDPNAGSYN-PWY and PWYO-1261 in DL, whereas PWY-7323, NAGLIPASYN-PWY, PWY-6467, PWY-5659, PWY-1269, P108-PWY, PWY-7539, PWY-6147, PWY-6700, PANTO-PWY, PWY-7199, P42-PWY, PANTOSYN-PWY, POLYISOPRENSYN-PWY, FOLSYN-PWY, GLUCONEO-PWY, PWY-6612, PWY-5695, RIBOSYN2-PWY, DTDPRHAMSYN-PWY, COA-PWY, PWY-6609, PWY0-1319, PWY-5667, PWY-5097, and PWY-5686 in PL yaks. Among the top 10 pathways, PWY0-1298, PWY0-781, PWY-5347, Argsynbsub-PWY, P4-PWY, Glycolysis-E-D, P23-PWY, Daplysinesyn-PWY, Udpnagsyn-PWY, and PWYO-1261 were notably higher in DL yaks, while PWY-7323, Naglipasyn-PWY, PWY-6467, PWY-5659, PWY-1269, P108-PWY, PWY-7539, PWY-6147, PWY-6700, and Panto-PWY were significantly higher in PL yaks (Figure 9a). Our results show that these pathways exhibited notably elevated levels and higher activity from 51 pathways. However, KEGG pathway analysis indicated 35 markedly different pathways, including biosynthesis of ansamycins, bacterial chemotaxis, flagellar assembly, sulfur relay system, limonene and pinene degradation, chloroalkane and chloroalkene degradation, linoleic acid metabolism, ABC transporters, nitrotoluene degradation, valine, leucine and isoleucine biosynthesis, porphyrin and chlorophyll metabolism, phosphonate and phosphinate metabolism, and a two-component system; these were prominent in DL yaks, while apoptosis, NOD-like receptor signaling pathway, oxidative phosphorylation, purine metabolism, protein digestion and absorption, tropane, piperidine and pyridine alkaloid biosynthesis, beta-alanine metabolism, peroxisome, riboflavin metabolism, DNA replication, pyrimidine metabolism, homologous recombination, cell cycle-caulobacter, carbon fixation in photosynthetic organisms, ubiquinone and other terpenoid-quinone biosynth, drug metabolism—other enzymes, zeatin biosynthesis, one carbon pool by folate, folate biosynthesis, lipopolysaccharide biosynthesis, streptomycin biosynthesis, and lipoic acid metabolism were prominent in PL yaks. Among them, the top 10 pathways, biosynthesis of ansamycins, bacteria chemotaxis, flagellar assembly, sulfur relay system, limonene and pinene degradation, chloroalkane and chloroalkene degradation, linoleic acid metabolism, ABC transporters, nitrotoluene degradation, and valine, leucine, and isoleucine biosynthesis, were significantly higher (*p* < 0.05) in DL yaks. Conversely, cell cycle-caulobacter, carbon fixation in photosynthetic organisms, ubiquinone and other terpenoid–quinone biosynthesis, drug metabolism—other enzymes, zeatin biosynthesis, one carbon pool by folate, folate biosynthesis, lipopolysaccharide biosynthesis, streptomycin biosynthesis, and lipoic acid metabolism were notably higher in PL yaks (Figure 9b). The KEGG pathway showed that these pathways exhibited notably higher levels and activity out of the 35 pathways.

## 4. Discussion

The gut microbiota mainly consists of bacteria (98%), followed by fungi (<0.1%) [44], which are not only responsible for digestion but also play an important role in protection against notorious pathogens (more than 80% of immune cells are found in the gut) [45]. Rumen microbes are especially integral in ruminants for efficient digestion and energy harvesting through the production of VFAs, fermenting sugar, and other carbohydrates [46]. The symbiotic relationship between ruminants and their microbiota is essential for nutrient utilization and reduction in methane emission and enables yaks to adapt to their ecological niche [47,48].

In the current study, we sequenced the rumen samples from plateau yaks with 14 (PL) and 15 (DL) ribs, obtaining 643,713 and 656,346 raw sequences and 611,934 and 622,814 filtered sequences in DL and PL animals, respectively. Alpha diversity had no valid difference between PL and DL Jiani animals, which is consistent with published results [49,50]. The dominating phyla in PL and DL yaks were Bacteroidota, Firmicutes_A, Firmicutes_C, and Firmicutes_D, which is in line with a previous study [37]. Different investigations reported that Firmicutes, proteobacteria, and Bacteroidetesare the predominant phyla in yaks’ intestinal tracts [51]. It is possible that the enhancement of Firmicutes and Bacteroides in the PL group enabled the efficient decomposition of lignin and cellulose and non-fibrous compounds [52,53]. These two groups produce B-group vitamins, which may have an anti-inflammatory influence and improve intestinal barrier function [54]. Another study found that Bacteroidota is essential for breaking complex carbohydrates in ruminants, generating energy through fermentation [28]

In the current study, cyanobacteria increased in PL yaks. This increase indicates their role in the digestion of fiber and in the absorption of nutrients. The elevation of cyanobacteria may indicate a unique adaptation to high-altitude areas, playing a role in nitrogen fixation and other metabolic processes for the better survival of yaks [28]. Therefore, the higher levels of Firmicutes_A (*p* < 0.05), Thermoplasma-tota (*p* < 0.05), Synergistota (*p* < 0.05), and Armatimonadota (*p* < 0.01) in DL yaks reflect differences in dietary intake or digestive efficiency. Firmicutes play an integral role in fermentative digestion, particularly in the breakdown of complex carbohydrates into SCFAs (short-chain fatty acids) [28]. Typically, the Firmicutes/Bacteroidetes rate is viewed as a significant pointer of flora composition [55], and microbiome shifts have been observed in individuals of different ages with varying Firmicutes/Bacteroidetes ratios [56]. The Bacteroidota/Firmicutes ratio differed between PL (3.04) and DL (2.35) ruminants, and six phyla showed marked differences between DL and PL yaks, suggesting differences in the microbiota structures of Jiani yaks.

At the genus level, the abundance of twenty-one genera differed significantly in Jiani yaks with different ribs. For instance, a previous study found increased *Limivicinus* in cattle fed with wheat during the stocker stage, influencing later animal performance [56,57]. The higher abundance of this genus in DL yaks may promote their growth. *Pyramidobacter* is a genus related to the degradation of toxic fluoroacetate from plants [58], which suggests that DL Jiani yaks may have a better ability to detoxify. *UBA71* is a genus of methanogenic archaea [59], and its higher abundance in DL yaks may contribute to rumen fermentation. The higher abundance of *Reyranella* in DL Jiani yaks may indicate better health status, as this genus is usually found in healthy animals. *Anaeroplasma* is typically found in healthy animals and is negatively related to inflammatory factors [60,61]. *Anaeroplasma* is well known for its role in the fermentation of carbohydrates [62], suggesting that PL yaks might be utilizing different substrates compared to DL yaks. The lower abundance of this genus in DL ruminants may increase inflammation levels in yaks.

The presence of thermoplasmatota and synergistota may suggest their interrelations with specialized metabolic pathways that facilitate digestion under specific dietary conditions. The genera such as *SFMI01*, *WRMH01*, and *Limivicinus*, which were significantly higher in DL yaks, play a role in specific metabolic processes in coordination with other gut microbiomes that help in digestion or immune-related functions. For example, *WRMH01* has been specifically linked to the digestion of fiber [62]. This indicates that the DL yaks have a more efficient microbial community for the processing of specific feed to which they are exposed. Conversely, the notable increase in genera like *UBA4334* and *Scatocola* in PL yaks indicates a different microbial strategy that may be more suited to their specific environment or dietary conditions. However, future studies can investigate the longitudinal assays that show how seasonal variations affect gut microbiota composition and the effect of different feeding strategies on the diversity of the gut microbiome. Exploration of functional capabilities using metagenomic approaches have identified different genera of great importance.

The findings of this study align with research indicating that yak gut microbiota is influenced by different factors like the diet, environment, health status, and genetics of the animals [31]. However, there remains a gap in understanding regarding how these microbial communities adapt to extreme environmental conditions typical of high-altitude regions; further studies can describe an interrelation between functional implications of these differences with regard to yak health and productivity.

The PICRUSt2 analysis indicated 51 significantly different metabolic pathways in two groups of yaks. Predominantly, pathways such as PWY0-781, PWY0-1298, PWY-5347, and others were enhanced in DL yaks. These pathways are responsible for different metabolic reactions, e.g., amino acid biosynthesis and metabolism of carbohydrates, which are critical for nutrient utilization and energy generation [63,64]. Conversely, metabolic pathways like NAGLIPASYN-PWY and PWY-7323 were upregulated in PL yaks, indicating potential differences in their metabolic capabilities or dietary adaptations.

Genes related to the biosynthesis of bacterial chemotaxis, ansamycins, and flagellar assembly in DL yaks were significantly increased. These pathways are essential for microbial motility and interaction with the host environment, designing a more dynamic microbiota capable of adapting to varying conditions [65]. In contrast, PL yaks exhibited higher levels of pathways associated with oxidative phosphorylation, apoptosis, and DNA replication, which may reflect differences in cellular processes or different stress responses. Previous studies highlighted the importance of rumen microbiota in ruminant survival [66,67]. For example, studies have indicated that variations in microbial communities significantly affect the metabolic pathway in the host. The metabolic pathways are influenced by both the diet and genetic makeup of the animal [68]. Pathways such as the super pathway of homolactic fermentation, glucose, and xylose degradation, chloroalkene and chloroalkane degradation, and pinene and limonene degradation were higher in DL Jaini yaks, which may contribute to rumen fermentation [69].

The results of the current study reinforce that different feeding plans are associated with anatomical variations that lead to a diversity of metabolic pathways within the gut environment [65]. This reflects the adaptability of yak microbiota to their environmental conditions, which is integral to their survival on the Qinghai–Tibet plateau. However, there is a need for further exploration to understand how selective breeding may have altered microbial capabilities compared to their wild counterparts.

## 5. Conclusions

This study elucidates the complex interactions between yaks with different rib structures (genetics) and the composition of gut microbiota. Our study indicates that yaks with 14- and 15-rib structures exhibit significant differences in microbial communities. Overall, 6 phyla and 21 genera differed significantly between the two groups with Bacteroidota and Firmicutes_A, dominating in yaks with 14-rib structures at the phylum level, while in yaks with 15-rib structures, Firmicutes_A showed higher relative abundance. This study underscores the importance of anatomical variations in shaping gut microbiota. Understanding these relationships can lead to better breeding strategies aimed at optimizing gut health in different husbandry conditions. Future studies should investigate these results by expanding the sample size and studying the influence of different environmental factors that cause microbial variations. Overall, our findings may provide novel insights into the future farming of yaks on the plateau.

## Figures and Tables

**Table 1:** Statistical analysis of sequences obtained from yaks with different ribs.

Samples	Input	Filtered	Denoised	Merged	Non-Chimeric	Non-Singleton
DL1	117,767	111,901	109,502	98,404	94,984	94,799
DL2	105,711	100,836	99,149	90,214	81,242	81,069
DL3	108,722	103,117	101,001	89,637	80,366	80,123
DL4	103,819	98,525	95,849	86,138	83,484	83,280
DL5	105,596	100,486	97,966	87,297	84,504	84,279
DL6	102,098	97,069	95,360	86,388	79,023	78,887
PL1	102,147	96,919	93,892	75,369	67,920	67,414
PL2	114,436	108,751	105,686	88,862	82,447	81,949
PL3	117,157	110,999	108,047	90,483	81,909	81,477
PL4	104,603	99,068	96,858	84,893	81,013	80,739
PL5	115,905	110,008	107,712	95,028	87,835	87,485
PL6	102,098	97,069	95,360	86,388	79,023	78,887

**Table 2:** Statistical analysis of annotated ASVs in different taxa.

Samples	Domain	Phylum	Class	Order	Family	Genus
DL1	38	2	80	48	470	785
DL2	18	0	39	43	259	353
DL3	24	1	51	36	238	506
DL4	44	0	43	53	438	767
DL5	31	1	63	48	370	645
DL6	17	0	35	46	227	382
PL1	45	1	58	66	377	938
PL2	39	0	88	69	327	982
PL3	54	1	56	51	375	953
PL4	37	0	56	69	378	848
PL5	35	2	61	54	307	682
PL6	9	0	31	22	201	298

**Table 3:** Statistical analysis of alpha diversity index in yaks with different ribs.

Sample	Chao1	Faith pd	Goods Coverage	Observed Species	Pielou_e	Shannon	Simpson
PL1	2578.435063	177.9602049	0.999036585	2573.4	0.869369371	9.849485164	0.996796838
PL2	2768.300767	178.4356119	0.997354902	2726.7	0.883354722	10.08167354	0.997965194
PL3	2523.677172	170.8505634	0.997637525	2488.5	0.864121407	9.748203389	0.997230552
PL4	2557.35822	172.4558957	0.998032572	2527.6	0.868535719	9.817537509	0.997148782
PL5	2225.421595	185.735405	0.997328358	2175.9	0.827317848	9.172796771	0.994368572
PL6	1113.989904	105.2390167	0.999183361	1100.5	0.754510713	7.623530424	0.982014854
DL1	2541.719479	169.0619051	0.997731212	2494.3	0.874597844	9.869327067	0.996916464
DL2	1316.166035	116.0392654	0.998836719	1299.1	0.767046622	7.93378748	0.987871152
DL3	1681.467705	151.0525443	0.998638415	1663	0.790611692	8.459203729	0.990012752
DL4	2494.898704	159.2174989	0.998313633	2466.4	0.883252138	9.9526533	0.99768237
DL5	2284.533438	160.0005751	0.998326125	2258.9	0.836061389	9.314895059	0.993691643
DL6	1268.09727	118.4587605	0.999041269	1254.5	0.792330395	8.155373126	0.990705739

## Data Availability

All raw sequence data from Jiani Yaks was deposited in the NCBI Sequence Read Archive database under accession number: PRJNA1095822.
